# Gender differences in sensitivity to the lenght of perioperative cold ischemia in donor myocardium

**DOI:** 10.1186/1749-8090-8-S1-O154

**Published:** 2013-09-11

**Authors:** M Smetana, J Pirk, A Lodererova, J Maluskova, O Szarszoi

**Affiliations:** 1 Department of Cardiovascular Surgery, Institut for Clinical and Experimental Medicine, Praque, Czech Republic; 2 Department of Pathology, Institut for Clinical and Experimental Medicine, Praque, Czech Republic

## Background

Many structural and metabolic functions of cardiovascular apparatus are essentially affected by gender. The experiments on animals have shown that female heart is more resistant to pathologic processes than male heart. The aim of our study was to find out the tolerance of human female and male graft to perioperative cold ischemia.

## Methods

The prospective study involved 35 consecutive patients undergoing heart transplantation from 9/2010 to 10/ 2011. Patients were divided into two groups: male graft (n = 20), female graft (n = 15). Troponin I as a marker of myocardial ischemia was measured at intervals: prior to collection of the donor, the recipient before sternotomy, 2h after transplantation, 1st postoperative day (POD), 3rd POD and one week after surgery. Moreover, apoptosis induced by heart ischemia was evaluated from biopsy specimens (before graft harvesting, 20 min after releasing of crossclamp, one week after transplantation) using bcl-2 and caspase 3.

## Results

We did not observe differences between groups in the level of troponin I 2h after transplantation, nevertheless troponin I was lower in patient with female graft at 1st POD (25.16 ± 4.75 vs. 18.79 ± 2.75); a week after transplantation we did not see any differences between groups. There were no differences in the expression of bcl-2 and caspase 3 before ischemia in both groups. Bcl-2 after surgery gradually increased in the male group and one week after transplantation was significantly higher compared to female group (1.41 ± 0.23 vs. 0.29 ± 0.16). Caspase 3 in the male group was stable during the reporting period, but one week after procedure in female hearts was significantly lower compared to male (5.35 ± 0.89 vs. 2.07 ± 0.67).

## Conclusions

Our results support hypothesis that the female myocardium is more resistant to cold heart ischemia and apoptosis has been shown to contribute to reduced loss of cardiomyocytes in female hearts. Study was supported by Grant IGA MZ NT/11269 – 5.

